# Refixation Saccades with Normal Gain Values: A Diagnostic Problem in the Video Head Impulse Test: A Case Report

**DOI:** 10.3389/fneur.2017.00081

**Published:** 2017-03-14

**Authors:** Leise Elisabeth Hviid Korsager, Christian Emil Faber, Jesper Hvass Schmidt, Jens Højberg Wanscher

**Affiliations:** ^1^Department of ENT Head and Neck Surgery, Odense University Hospital, Denmark; ^2^Department of Audiology, Odense University Hospital, Denmark

**Keywords:** video head impulse test, cochlear implant, vestibular function, vestibulo-ocular reflex, refixation saccades

## Abstract

Refixation saccades with normal gain value occur more frequently with increasing age. The phenomenon has also been observed in different vestibular disorders. In this case, we present a young male with normal gain value and refixation saccades tested with the video head impulse test (vHIT) the day after his cochlear implantation. One month after surgery, refixation saccades were no longer present. This suggests that refixation saccades can occur as a result of temporary pathology such as surgery. Refixation saccades with normal gain values might reflect a partial deficit in the vestibulo-ocular reflex. However, this partial deficit is in conflict with the current way of interpreting vHIT results in which the vestibular function is classified as either normal or pathological based only on the gain value. Refixation saccades, which are evident signs of vestibulopathy, are not considered in the evaluation. A new way of interpreting the vHIT based on the saccades must be considered.

## Introduction

The video head impulse test (vHIT) is a new diagnostic tool to investigate the function of the semicircular canals in the vestibular organ. It is based on the vestibulo-ocular reflex (VOR), which ensures that the eyes can fix a target despite head movement.

The semicircular canals are activated by head rotation and their impulses are transferred *via* the vestibular nerve endings located in the vestibular nuclei in the brainstem. Fibers cross to the contralateral nucleus abducens and send signals to the eye muscles. This results in eye movements that are in the direction opposite to the head movement.

The vHIT evaluates semicircular canal function by calculating a VOR gain value and presenting the VOR in a diagram. Covert and overt saccades can be detected on the diagram.

Currently the interpretation of a vHIT result is based purely on the gain value, which is used to determine whether the vestibular function is classified as either normal or pathological. However, this can lead to diagnostic problems. The method by which to interpret a gain value in the normal range combined with refixation saccades needs to be clarified as saccades are signs of evident vestibulopathy (1).

Refixation saccades are a result of impaired VOR (1). They are defined by an increased eye acceleration >4,000°/s and are visually identified by the vHIT examiner (2).

## Case

**Figure 1:**
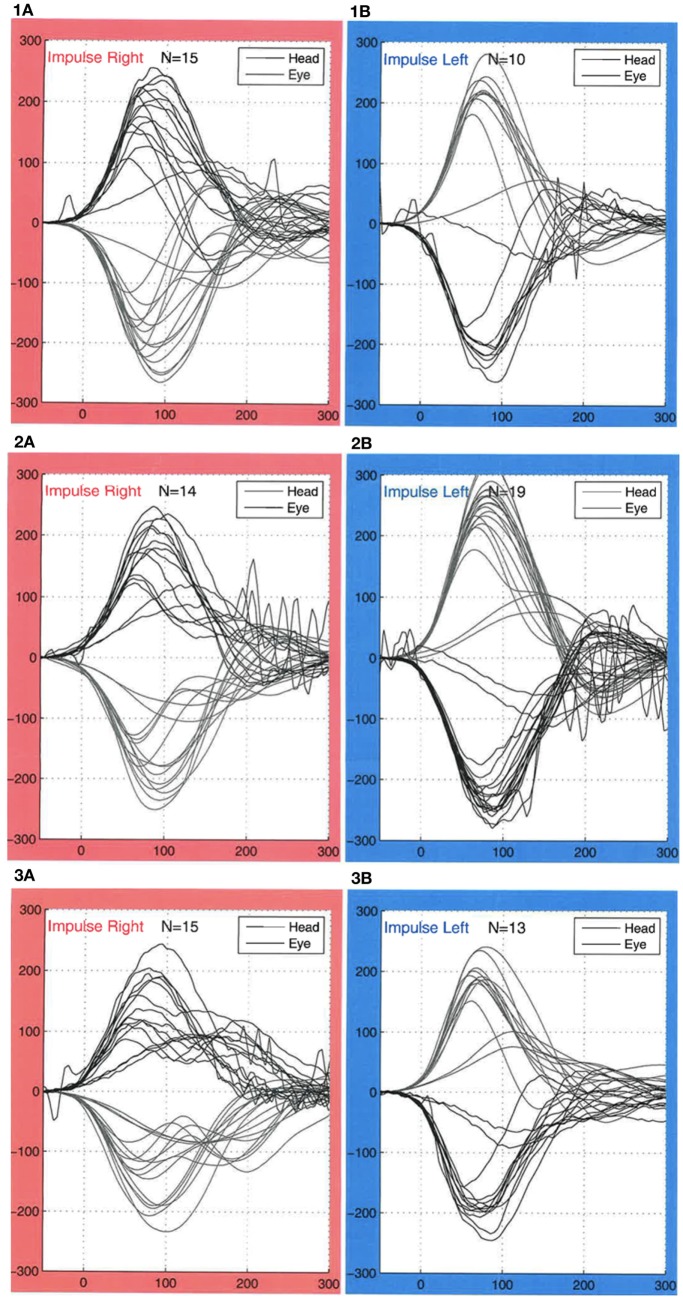
**Results of video head impulse video head impulse test (vHIT) testing**. For right-sided impulses (red boxes), the lower lines indicate the head movements, and the upper lines indicate eyes movements. For left-sided impulses (blue boxes), the upper lines indicate head movements, and the lower lines indicate eyes movement. (1a,1b) vHIT obtained before the patient cochlear implantation. Gain values were within normal, and no refixation saccades were observed. (2a,2b) vHIT from the day after the cochlear implantation. Gain values were within normal ranges. Refixation saccades with a peak velocity of maximum of 100°/s were observed bilaterally. The examiner observed the saccades on the computer screen during examination and were therefore not the result of artifacts. (3a,3b) Gain values were still within normal, and no refixation saccades were observed one month after the surgery.

A 38-year-old man was scheduled for bilateral implantation with Mid-Scala electrodes from Advanced Bionics (Advanced Bionics, Valencia, CA, USA). The patient had poor hearing since childhood but no history of vestibular disease, dizziness, or instability. Prior to surgery, a vHIT was conducted using the EyeSeeCam (Interacoustics a/s, Denmark). Gain values were within the normal range (1.02 ± 0.06 on the right side and 0.94 ± 0.27 on the left side). Asymmetry was 3% between the left and right ears, and no saccades occurred (Figure 1).

The day after surgery, the patient was feeling well and had no complaints of dizziness or balance problems. A new vHIT was conducted. Gain values of 1.02 ± 0.04 from the right ear and 0.90 ± 0.15 from the left ear were still within normal range. Asymmetry was 5% between left and right ears. However, at that time, refixation saccades occurred bilaterally. They had a peak velocity of maximum 100°/s and occurred between 180 and 400 ms. The saccades were also visible by naked eye during head impulses and thus were not the result of artifacts.

One month after surgery, the implants were activated. A vHIT was then conducted. Gain values were still within the normal range with readings of 1.03 ± 0.05 from the right ear and 1.01 ± 0.06 from the left ear. No saccades were seen at this point.

## Discussion

Refixation saccades with normal gain values have been reported to occur more frequently with increasing age (2–4).

Video head impulse test was used to test the horizontal semicircular canal in 212 healthy subjects who had no history of vestibular disease (2). Refixation saccades with normal gain values were detected in 52 subjects and were significantly more common after the age of 71 years. Gain values were significantly lower when refixation saccades occurred but still within the normal range. Because the subjects did not suffer from vestibular disease, only aging seems to explain the refixation saccades.

Aging can alter the vestibular system in several ways. Loss of vestibular hair cells and age-related changes such as deformation of the cilia can alter the VOR. Loss of neurons within the vestibular system should also be considered. These slight reductions in VOR gain with increasing age may trigger refixation saccades. It has also been proposed that the saccade-generating mechanism in elderly adults may be impaired relative to younger adults.

However, refixation saccades with normal gain values have also been reported in younger populations. Using vHIT, Perez-Fernandez and Eza-Nunez examined 623 patients with vertigo and dizziness (4). In the final population, he included patients with gain values > 0.8 with unilateral refixation saccades. The final population consisted of 36 patients: 29 patients had Ménière’s disease, 4 had post neuritis vestibularis, 1 had vestibular associated otosclerosis, 1 had post-concussion, and 1 was a vestibular schwannoma patient. This suggests that refixation saccades may not only be a matter of aging but can also be a result of vestibular pathology. All of the above mentioned vestibular diseases can damage either the vestibular organ and/or nerve. This type of damage can impair the VOR thus leading to saccades and low gain values. With time, central compensation will occur resulting in normal gain values. However, since the VOR is still impaired, saccades will still be generated.

In our case, the patient was 38 years old; therefore, the refixation saccades were not a result of aging. The patient did not suffer from any vestibular disorder, and since the saccades occurred immediately after the surgery, it seems that the surgery most likely caused the saccades. They disappeared within one month, and we concluded that they can be temporary. Several mechanisms can be proposed: (i) loss of intracochlear fluid may affect the hair cells’ capability to create excitatory signals, which lead to VOR suppression and subsequent saccades and (ii) edema around the nerve may affect its ability to generate action potentials.

In a previous study, we suggested that the refixation saccades might be a better predictor of the vestibular function than gain value (5). The gain value is known to vary for a number of reasons, whereas the occurrence of saccades seems more reliable. A single cut off in gain value for normal VOR function makes it difficult to distinguish between partial VOR gain deficits such as those due to aging or vestibular pathology (6). Furthermore, to a variable degree, refixation saccades seem dependent on peripheral VOR gain for recovery from vestibulopathy. In a study with a patient recovering from vestibular neuritis, Schubert et al. detected reduced frequency, amplitude, velocity, and latency of the refixation saccades as the VOR gain partially recovered. The reduced latency suggests that the brain learned to interject the refixation saccades during recovery (7). Using frequency, amplitude, velocity, and latency of refixation saccades may be a new way to predict vestibular function.

## Conclusion

Refixation saccades with gain values in the normal range occurred more frequently with increasing age but are also reported following different vestibular disorders such as Ménière’s Disease. From this case, we conclude that the phenomenon can be present after cochlear implantation and can be temporary.

Refixation saccades might reflect a partial dysfunction of the VOR. Interpretation of the phenomenon is in conflict with the current way of interpreting the vHIT, which is only based on the gain value and is used to classify vestibular function as either normal or pathological.

A new way of interpreting the vHIT based on refixation saccades must now be considered.

## Ethics Statement

The authors assert that all procedures contributing to this work comply with the ethical standards of the Regional Committees on Health Research Ethics for Southern Denmark and with the Helsinki Declaration of 1975, as revised in 2008. Informed consent was obtained from the included subject.

## Author Contributions

The authors declare given substantial contributions to the conception, acquisition, analysis, and interpretation of the manuscript. All authors have revised the manuscript critically for important intellectual content and declared the final approval of the version to be published, and agreed to be accountable for all aspects of the work in ensuring that questions related to the accuracy or integrity of any part of the work are appropriately investigated and resolved by all authors.

## Conflict of Interest Statement

The authors declare that the research was conducted in the absence of any commercial or financial relationships that could be construed as a potential conflict of interest.
